# Genetic Compatibility of Reassortants between Avian H5N1 and H9N2 Influenza Viruses with Higher Pathogenicity in Mammals

**DOI:** 10.1128/JVI.01969-18

**Published:** 2019-02-05

**Authors:** Yasuha Arai, Madiha S. Ibrahim, Emad M. Elgendy, Tomo Daidoji, Takao Ono, Yasuo Suzuki, Takaaki Nakaya, Kazuhiko Matsumoto, Yohei Watanabe

**Affiliations:** a Department of Infectious Diseases, Kyoto Prefectural University of Medicine, Kyoto, Japan; b Department of Microbiology and Immunology, Faculty of Veterinary Medicine, Damanhour University, Damanhour, Egypt; c The Institute of Scientific and Industrial Research, Osaka University, Osaka, Japan; d College of Life and Health Sciences, Chubu University, Aichi, Japan; e CREST, Japan Science and Technology Agency, Saitama, Japan; St. Jude Children’s Research Hospital

**Keywords:** H5N1, H9N2, influenza reassortants, pathogenicity, public health risk

## Abstract

The cocirculation of H5N1 and H9N2 avian influenza viruses in birds in Egypt provides reassortment opportunities between these two viruses. However, little is known about the emergence potential of reassortants derived from Egyptian H5N1 and H9N2 viruses and about the biological properties of such reassortants. To evaluate the potential public health risk of reassortants of these viruses, we used reverse genetics to generate the 63 possible reassortants derived from contemporary Egyptian H5N1 and H9N2 viruses, containing the H5N1 surface gene segments and combinations of the H5N1 and H9N2 internal gene segments, and analyzed their genetic compatibility, replication ability, and virulence in mice. Genes in the reassortants showed remarkably high compatibility. The replication of most reassortants was higher than the parental H5N1 virus in human cells. Six reassortants were thought to emerge in birds under neutral or positive selective pressure, and four of them had higher pathogenicity *in vivo* than the parental H5N1 and H9N2 viruses. Our results indicated that H5N1-H9N2 reassortants could be transmitted efficiently to mammals with significant public health risk if they emerge in Egypt, although the viruses might not emerge frequently in birds.

**IMPORTANCE** Close interaction between avian influenza (AI) viruses and humans in Egypt appears to have resulted in many of the worldwide cases of human infections by both H5N1 and H9N2 AI viruses. Egypt is regarded as a hot spot of AI virus evolution. Although no natural reassortant of H5N1 and H9N2 AI viruses has been reported so far, their cocirculation in Egypt may allow emergence of reassortants that may present a significant public health risk. Using reverse genetics, we report here the first comprehensive data showing that H5N1-N9N2 reassortants have fairly high genetic compatibility and possibly higher pathogenicity in mammals, including humans, than the parental viruses. Our results provide insight into the emergence potential of avian H5N1-H9N2 reassortants that may pose a high public health risk.

## Abstract (precis)

Close interaction between avian influenza (AI) viruses and humans in Egypt appears to have resulted in many of the worldwide cases of human infections by both H5N1 and H9N2 AI viruses. Egypt is regarded as a hot spot of AI virus evolution. Although no natural reassortant of H5N1 and H9N2 AI viruses has been reported so far, their cocirculation in Egypt may allow emergence of reassortants that may present a significant public health risk. Using reverse genetics, we report here the first comprehensive data showing that H5N1-N9N2 reassortants have fairly high genetic compatibility and possibly higher pathogenicity in mammals, including humans, than the parental viruses. Our results provide insight into the emergence potential of avian H5N1-H9N2 reassortants that may pose a high public health risk.

## INTRODUCTION

The influenza A virus genome consists of eight RNA gene segments. This genome arrangement allows the reassortment of gene segments between two influenza viruses that coinfect a single host cell. Reassortment is the main mechanism for influenza virus adaptation to new hosts by the generation of novel viral genotypes. In addition, point mutations, mostly in the viral polymerase and hemagglutinin (HA) genes, are critical for efficient viral replication in new hosts. The most well-known human adaptation mutation is the polymerase subunit PB2-E627K substitution that enables efficient replication of avian influenza (AI) viruses at 33°C, which mimics the temperature in the human upper airway (1, 2). AI virus HA also must change its binding preference from avian-type α2,3-linked sialic acid (α2,3 Sia) to human-type α2,6-linked sialic acid (α2,6 Sia) for efficient infection of humans (3, 4).

Since its emergence in China in 1996 (5), highly pathogenic avian H5N1 influenza viruses have become endemic in birds in several geographic areas, including China and Egypt. H5N1 viruses can be transmitted directly from birds to humans, if there is close interaction, and have caused 860 confirmed human infections with 52.8% mortality (as of 21 September 2018, according to the World Health Organization [WHO]). Egypt has unexpectedly had more human H5N1 infections than any other country, with about 66.2% of the cases worldwide since 2009 (as of 2 March 2018, according to the WHO). This implied appreciably closer interaction between H5N1 viruses and humans in Egypt than in other geographic areas and/or virological properties that are not yet understood (6). The epidemiological situation has been further complicated by the start of endemic circulation of the H9N2 G1 lineage of subclade B AI viruses (H9N2 G1-B) in birds (7, 8), with four clinically mild human H9N2 infection cases in Egypt (FluTrackers). A seroepidemiological study has indicated that human H9N2 infections are more prevalent in Egypt than has been reported (9).

A number of zoonotic influenza viruses (e.g., the H5N1 Gs/GD lineage, and H7N9, H10N8, H5N8, and H5N6 viruses) have emerged with reassortment of some H9N2-G1 internal genes (10–13). This indicated that H9N2 G1 viruses could be a source of influenza virus internal genes for generating novel viral reassortants (10, 14). Cocirculation of the H5N1 and H9N2 G1-B subtypes in Egypt provides opportunities for reassortment of the genes of these viruses. No natural reassortant of these two subtypes has been reported in Egypt thus far. However, continuous cocirculation may allow emergence of reassortant viruses that could pose a serious public health threat. Studies have reported cocirculation of H5N1 and H9N2 subtypes in the same geographic areas, in particular in poultry farms and some bird species in Egypt (7, 8). However, there have been few large-scale studies of the possible emergence of such influenza virus reassortants in Egypt, although a few experimental studies have rescued reassortant viruses of the two subtypes that have been isolated in Egypt, which indicated some level of genetic compatibility between the genes of these viruses (15, 16).

In this study, we wanted to investigate the public health risk of possible reassortants derived from the H5N1 and H9N2 viruses that currently cocirculate in Egypt. Therefore, we used reverse genetics to systematically generate a set of reassortants between the H5N1 and H9N2 viruses isolated in Egypt. We investigated the phenotypes of reassortants containing the H5N1 HA and NA surface gene segments and one of the 63 (2^6^-1) combinations of the six internal gene segments of the H5N1 and H9N2 viruses. The comprehensive *in vitro* and *in vivo* analyses of the reassortants reported here show remarkably high compatibility of the gene segments of the contemporary H5N1 and H9N2 influenza viruses that have been isolated in Egypt. These data provide insight into the potential future emergence of influenza viruses in nature with high infectivity and pathogenicity in mammalian species, including humans.

## RESULTS

### Recovery of reassortants derived from contemporary H5N1 and H9N2 viruses in Egypt.

**FIG 1:**
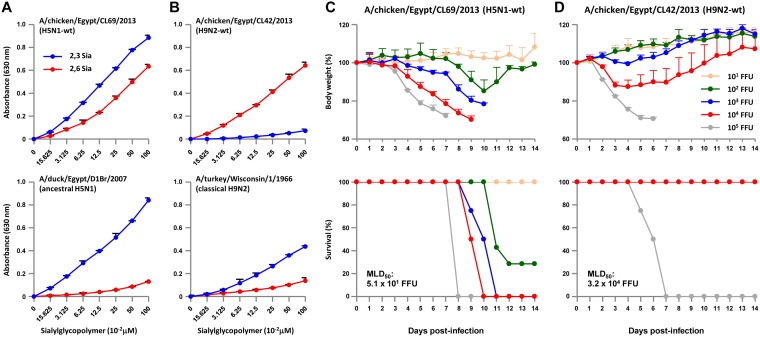
Infectivity and virulence of H5N1-wt and H9N2-wt viruses. (A and B) Binding of H5N1-wt (A) and H9N2-wt (B) to α2,3 sialylglycopolymers (blue) and α2,6 sialylglycopolymers (red). A/duck/Egypt/D1Br/2007 and A/turkey/Wisconsin/1/1966 are ancestral H5N1 clade 2.2.1 and classical H9N2 strains, respectively. Each data point reflects the mean ± the SD of three independent experiments. (C and D) Virulence in mice infected with the H5N1-wt (C) and H9N2-wt viruses (D). Five-week-old BALB/c mice (five mice per group) were inoculated intranasally with serial 10-fold dilutions of the viruses. The body weights of infected mice were monitored for 14 dpi. The mean ± the SD of the percentage of the initial body weight for each group of mice is shown. Survival was calculated, including mice that were sacrificed after they had lost more than 30% of their body weight.

During 2011 to 2013, we carried out an epidemiological study of influenza viruses in Egypt and isolated two viruses, A/chicken/Egypt/CL69/2013 (H5N1) and A/chicken/Egypt/CL42/2013 (H9N2). As reported by others (8), phylogenetic analyses of the eight gene segments of these viruses indicated cocirculation of H5N1 and H9N2 viruses in Egypt and showed that A/chicken/Egypt/CL69/2013 (H5N1) and A/chicken/Egypt/CL42/2013 (H9N2) are representative strains of contemporary H5N1 clade 2.2.1.2 and H9N2 G1-B influenza viruses isolated in Egypt (see Fig. S1 and Fig. S2 in the supplemental material). The H5N1 clade and H9N2 lineage are unique to this area (8, 17). A/chicken/Egypt/CL69/2013 (H5N1) and A/chicken/Egypt/CL42/2013 (H9N2) are referred to here as CL69 and CL42, or as H5N1-wt and H9N2-wt viruses, respectively. To generate a set of reassortants for this study, we established a plasmid-based reverse-genetics system for generating recombinant viruses from parental H5N1 and H9N2 viruses. Receptor binding assays showed that both CL69 and CL42 have acquired increased binding affinity to α2,6 Sia compared to ancestral clade 2.2.1 and classical H9N2 strains, respectively (Fig. 1A and B), implying an increased avian-to-human infection potential of both subtypes in Egypt. However, CL69 and CL42 showed distinct virulence in mice (Fig. 1C and D). CL69 was highly virulent in mice with a 50% mouse lethal dose (MLD_50_) of 5.1 × 10^1^ focus-forming units (FFU) due to the presence of a multibasic cleavage site in the H5N1 HA (1). In contrast, CL42 was avirulent in mice due to a monobasic cleavage site in the H9N2 HA (18, 19), with an MLD_50_ of 3.2 × 10^4^ FFU, which was >600-fold more than the H5N1-wt MLD_50_. This indicated higher priority in studying reassortants containing the H5N1 HA and NA surface gene segments and combinations of the H5N1 and H9N2 internal gene segments for public health concerns (see Discussion). In fact, reassortment of H9N2 internal genes with another influenza virus subtype has led to emergence of zoonotic reassortants (10–13). Therefore, the recombinant viruses generated for this study were the 63 possible reassortants of Egyptian H5N1 and H9N2 viruses: each reassortant contained the H5N1 HA and NA surface gene segments and one of the 63 combinations of the H5N1 and H9N2 internal gene segments.

**FIG 2:**
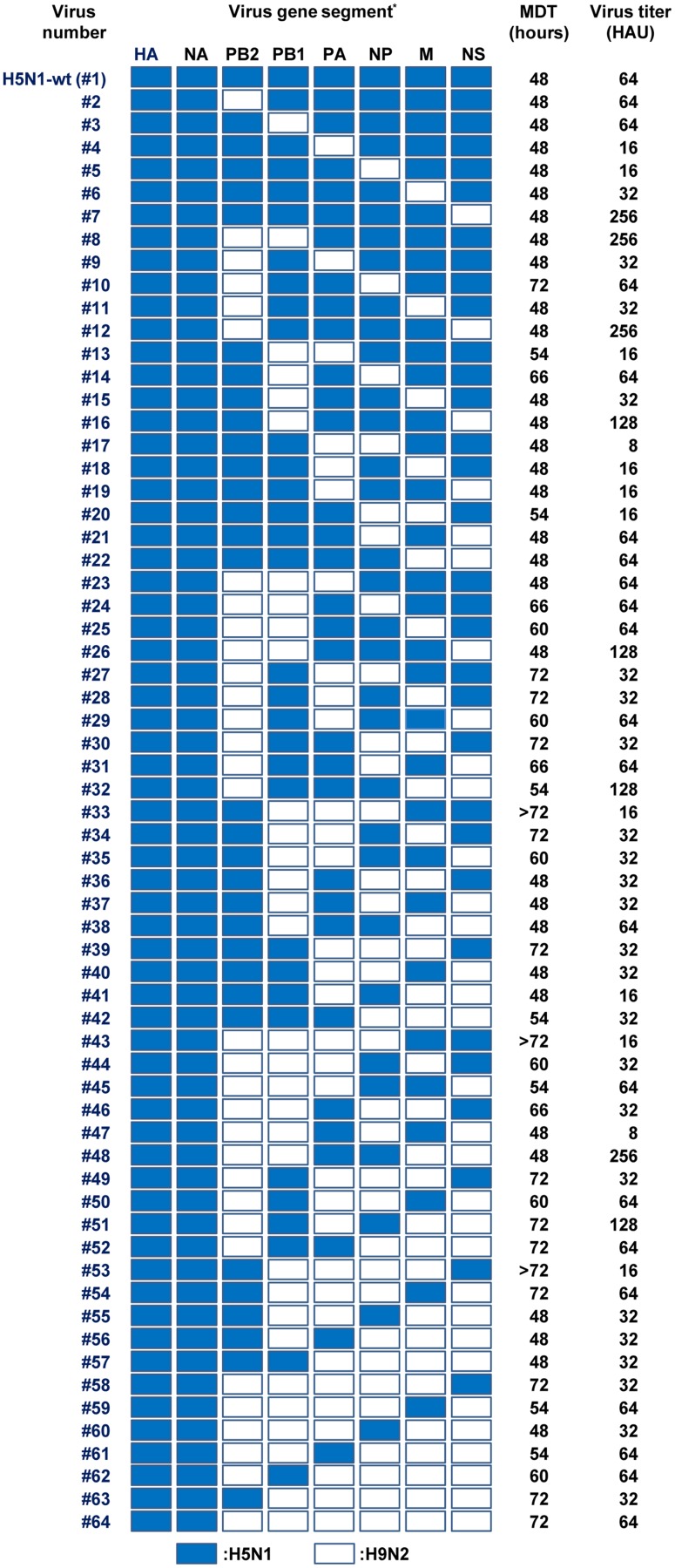
Properties of reassortants with H5N1 HA and NA surface genes and six internal genes from H5N1 and H9N2 viruses. The eight gene segments are indicated at the top of each column. Blue denotes an H5N1 gene segment and white denotes an H9N2 gene segment. The mean death time (MDT) was calculated based on egg death after inoculation with cell culture supernatants at 96 h posttransfection with plasmid DNAs. Virus stocks were prepared after one or two passages, and their titers were assayed as HAU by hemagglutination assays.

Virus recovery from the allantoic fluid of embryonated eggs inoculated with transfected cell culture supernatants was determined by hemagglutination assays. The virus titers in the allantoic fluids were >16 hemagglutination units (HAU) for H5N1-wt virus and all 63 reassortants, with a wide range of mean death times (Fig. 2), indicating a high degree of compatibility between the gene segments of H5N1 and H9N2 viruses and a broad spectrum of phenotypes.

### Replication of reassortants *in vitro*.

**FIG 3:**
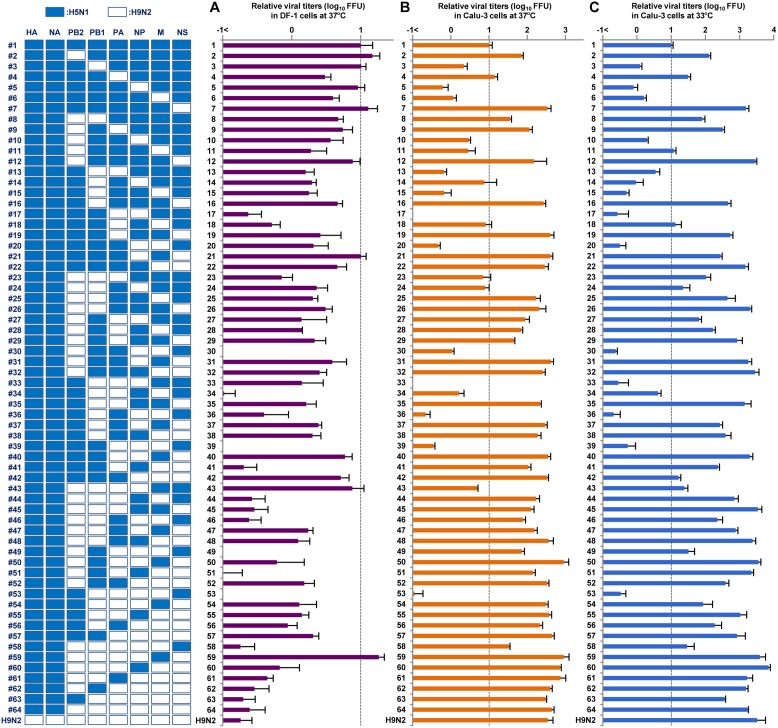
Replication of reassortants with H5N1 HA and NA surface genes and six internal genes from H5N1 and H9N2 viruses in avian and human cells. DF-1 (A) and Calu-3 (B and C) cells were infected with the indicated viruses at MOIs of 0.003 and 0.03, respectively, and cultured at 37°C for 48 hpi (A and B) or 33°C for 72 hpi (C). Progeny viruses were collected, and virus titers were determined by focus-forming assays. The titers were calculated relative to that of H5N1-wt. Each data point indicates the mean ± the SD of three independent experiments.

We next investigated the replication of H5N1, H9N2 and the 63 reassortants in human and avian cells. Chicken fibroblast (DF-1) cells and human bronchial epithelial (Calu-3) cells were infected with recombinant CL69 (H5N1-wt), CL42 (H9N2-wt), or one of the reassortants: DF-1 cells were infected at a multiplicity of infection (MOI) of 0.003, and Calu-3 cells were infected at an MOI of 0.03. After incubation at 37 or 33°C, virus titers were assayed as FFU at 48 or 72 h postinfection (hpi), as noted. In both cell types, replication of H9N2-wt was identical to that of the #64 virus, which carried HA and NA genes from H5N1-wt and all six internal genes from H9N2-wt (Fig. 3), indicating that *in vitro* replication was associated with the internal genes, but not with the surface genes.

At 37°C in DF-1 cells, replication of the H9N2-wt virus was 55.1-fold lower than that of the H5N1-wt (#1 virus) (*P* < 0.01), even with addition of the appropriate concentration of trypsin in the cell cultures for H9N2-wt growth (Fig. 3A). Although replication of most reassortants (55 of 63) was lower (i.e., up to 100-fold less) than that of H5N1-wt (*P* < 0.01), the replication of several reassortants (#2, #3, #5, #7, #12, #21, and #43 viruses) was at levels comparable to that of H5N1-wt (*P* > 0.05). An exception was the #59 virus that contained the H5N1 M gene segment in the H9N2 internal gene background and had significantly higher replication than that of H5N1-wt (*P* < 0.01). Among the reassortants, the 31 reassortants containing the H5N1 M gene (e.g., #2, #3, #5, #7, #12, #21, #43, and #59 viruses) retained substantial levels of replication in avian cells (an average of less than half that of H5N1-wt), whereas the remaining 32 reassortants containing the H9N2 M gene had appreciably attenuated replication (an average of 10.0-fold lower than that of H5N1-wt). This suggested that the H5N1 M gene segment provided a replication advantage for the reassortants in avian cells.

In contrast, replication of H9N2-wt in Calu-3 cells at 37°C was 34.3-fold higher than that of H5N1-wt (*P* < 0.01) (Fig. 3B). The replication of a majority of the reassortants (42 of 63) was an average of 28.5-fold higher than that of H5N1-wt (*P* < 0.01). In particular, replication of most reassortants containing the H9N2 NS gene segment (29 of 32; e.g., #7, #12, #19, #22, and #26 viruses) was an average of 34.6-fold higher than that of H5N1-wt (*P* < 0.01). Conversely, the replication of most reassortants containing the H5N1 NS gene segment (29 of 32; e.g., #17, #20, #33, #36, #39, #53, and #58 viruses) was up to 100-fold less than that H9N2-wt (*P* < 0.01). These data indicated that the H9N2 NS gene and the H5N1 NS gene acted to enhance or impair, respectively, the replication of reassortants in human cells regardless of the other internal genes in those reassortants. In addition, replication of reassortants containing the H9N2 PB2 gene segment tended to be higher in Calu-3 cells than that of H5N1-wt. Excluding reassortants with the H9N2 NS gene segment that masked the effects of other genes because of its strong effect, replication of most reassortants containing the H9N2 PB2 gene segment (10 of 16; e.g., #2, #8, and #9 viruses) was an average of 9.0-fold higher than that of H5N1-wt (*P* < 0.01). Similar replication patterns were observed in human cells at 33°C (Fig. 3C), except that the differences were more prominent than at 37°C. At 33°C, replication of many reassortants (46 of 63) was an average of 113.9-fold higher than that of H5N1-wt (*P* < 0.01), despite the gene segments in these reassortants being from both AI viruses.

Significant genetic incompatibility was observed in human cells between H5N1 and H9N2 gene segments, which was seen in the replication of reassortants containing the H5N1 PB2 gene segment and one or both of the H9N2 NP and M gene segments (Fig. 3B and C). Among the reassortants that did not contain the H9N2 NS gene segment that masked the effects of other genes, replication of most reassortants containing the H5N1 PB2 gene segment and either the heterologous H9N2 NP or M gene segment (5 of 8; e.g., #5, #17, and #33 viruses) or both the heterologous H9N2 NP and M gene segments (4 of 4; e.g., #20, #36, and #53 viruses) was less than 10% of that of H5N1-wt (*P* < 0.01). In contrast, replication of most reassortants containing the H9N2 PB2 gene segment and either the homologous H9N2 NP or M gene segment (7 of 8; e.g., #11, #24, and #25 viruses) or both the homologous H9N2 NP and M gene segments (3 of 4; e.g., #46, #49, and #58 viruses) was more than 100% of that of H5N1-wt.

### *In vitro* polymerase activity of the reassortants.

**FIG 4:**
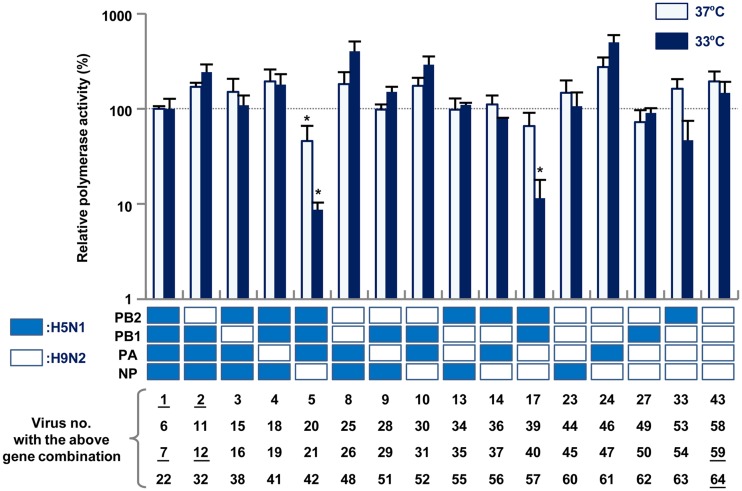
Viral polymerase activity in human cells infected with H5N1-H9N2 reassortants. 293T cells were transfected with plasmids expressing a combination of the PB2, PB1, PA, and NP genes from H5N1 or H9N2 viruses, a human polymerase I-driven plasmid expressing a reporter gene, and a *Renilla* luciferase-expressing plasmid as an internal control. After 24 h of incubation at 33 or 37°C, the luciferase activity of each sample was measured and normalized to the internal *Renilla* luciferase activity. The data were expressed relative to the results for H5N1-wt. Each data point represents the mean ± the SD of three independent experiments. Asterisks indicate relative polymerase activity significantly lower than that of a polymerase complex consisting of PB2, PB1, PA, and NP genes from the H5N1 virus (ANOVA with Tukey’s multiple-comparison test [*, *P < *0.01]). The number of the virus containing each corresponding gene combination is shown below the appropriate column, with the number of the viruses selected for further characterization underlined.

To study the mechanism(s) underlying the replication differences of the reassortants, the polymerase activity of 16 combinations of the polymerase and nucleoprotein genes (PB2, PB1, PA, and NP) from H5N1 and H9N2 viruses was determined by minigenome assays in human 293T cells. The H5N1-wt and H9N2-wt polymerases had similar activities at both 37 and 33°C (Fig. 4). Most of the reassortant combinations had a polymerase activity similar to that of H5N1-wt. These results suggested that other internal genes (e.g., M and NS) that were not included in these minigenome assays accounted for the differences in replication of the reassortants carrying H5N1 surface genes in human cells. However, two of the four combinations of the H5N1 PB2 and H9N2 NP genes had markedly reduced polymerase activity of about 10% that of H5N1-wt, especially at 33°C (*P* < 0.01). This indicated that the decreased polymerase activity was due to genetic incompatibility between H5N1 PB2 and H9N2 NP that attenuated the replication of reassortants containing H5N1 PB2 and H9N2 NP (e.g., #5, #17, and #33 viruses) in human cells (Fig. 3B and C).

### Growth kinetics of the reassortants *in vitro*.

**FIG 5:**
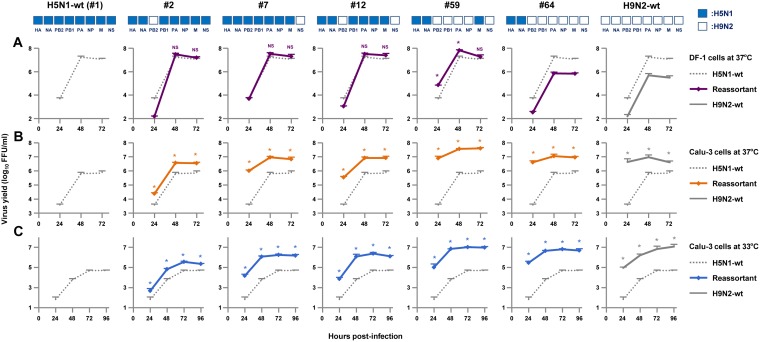
Growth kinetics of H5N1-H9N2 reassortants in avian and human cells. DF-1 (A) and Calu-3 cells (B and C) were infected with the indicated viruses at MOIs of 0.003 or 0.03, respectively, and incubated at 37 (A and B) or 33°C (C). At the indicated times postinfection, virus titers were determined by focus-forming assays. Each data point represents the mean ± the SD of three independent experiments. Asterisks indicate virus titers significantly higher than that of H5N1-wt virus (ANOVA with Tukey’s multiple-comparison test [*, *P < *0.01]). NS, no statistical significance.

Cocirculation of H5N1 and H9N2 viruses in Egyptian poultry provides a potential scenario for the emergence of AI reassortants, making virus transmission from birds to humans a public health concern. Among the 63 possible reassortant genotypes, replication of the #2, #7, and #12 viruses was at comparable levels to that of H5N1-wt in avian cells and was higher than that of H5N1-wt in human cells (Fig. 3). Moreover, replication of the #59 virus was significantly higher than that of H5N1-wt in both avian and human cells. These reassortant viruses contained the H5N1 M gene and the H9N2 PB2 and/or NS gene (Fig. 5, upper panel), which may enable bird-to-human virus transmission with high efficacy. Therefore, we selected these four reassortants for further study. In addition, the H5N1-wt, H9N2-wt, and #64 viruses were included in this study for comparison.

To investigate the growth kinetics of the reassortants, DF-1 and Calu-3 cells were infected with H5N1-wt, H9N2-wt, or one of the selected reassortants, and viral growth kinetics was assayed up to 72 hpi at 37°C and up to 96 hpi at 33°C. The results were in agreement with the virus titers at 48 and 72 hpi (Fig. 3). In DF-1 cells at 37°C, the #2, #7, and #12 viruses produced progeny virus titers comparable to that of H5N1-wt, and the #59 virus produced a higher progeny virus titer than H5N1-wt (Fig. 5A). However, the #64 virus, that was included as a control, produced a lower progeny virus yield than H5N1-wt, which was comparable to that of H9N2-wt. In contrast, in Calu-3 cells all the reassortants produced significantly higher progeny virus titers than H5N1-wt at all time points at 37°C (Fig. 5B), with replication levels comparable to H9N2-wt. In particular, replication of the #7, #59, and #64 viruses was much higher than that of H5N1-wt at early times postinfection (24 hpi), with more prominent differences at 33°C, i.e., an average of 200-fold higher at 37°C and an average of 440-fold higher at 33°C (Fig. 5C).

In agreement with their H5N1-H9N2 hybrid phenotypes, these data indicated that replication of the #2, #7, #12, and #59 viruses was (i) at levels comparable to or higher than that of H5N1-wt in avian cells, (ii) higher than that of H5N1-wt in human cells, and (iii) at similar levels to H9N2-wt. These reassortants were completely sequenced, and no mutations were found after they were selected as seed viruses or after they replicated in the cultured cells and mice in this study. Therefore, the phenotypes of the reassortant viruses in this study were the result of the intrinsic properties of the components of the reassortants and/or of their set of gene segments.

### Pathogenicity of the reassortants in mice *in vivo*.

**FIG 6:**
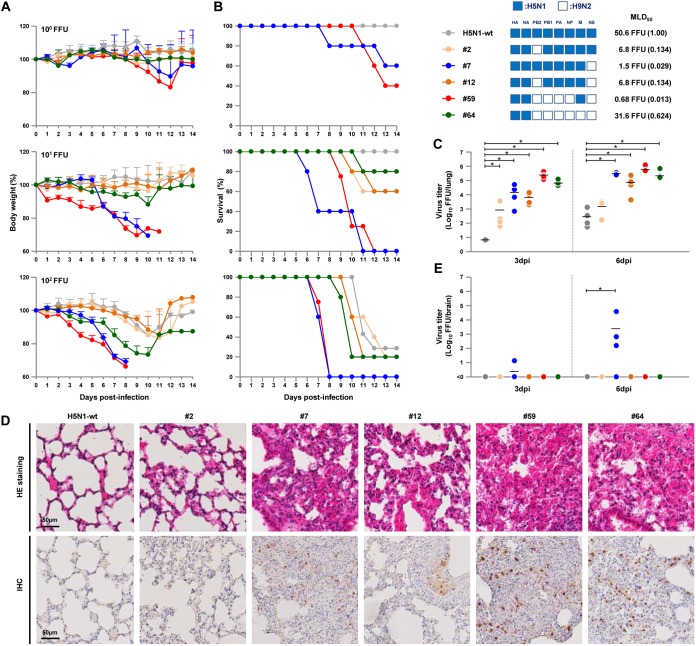
Body weights and survival of mice infected with H5N1-H9N2 reassortants. Five-week-old BALB/c mice (five mice per group) were inoculated intranasally with serial 10-fold dilutions of the indicated viruses. (A) The body weights of the infected mice were monitored for 14 dpi. The mean ± the SD of the percentage of the initial body weight for each group of mice is shown. (B) Survival of the infected mice. Survival was calculated including mice that were sacrificed after they had lost more than 30% of their body weight. MLD_50_ values of the viruses are shown, with each value relative to H5N1-wt in parenthesis. (C and E) Virus titers in the lungs (C) and brains (E) of mice infected with 10 FFU of the indicated viruses at 3 and 6 dpi. Each symbol marks the titer in an individual mouse. Asterisks indicate virus titers significantly higher than those of H5N1-wt virus (ANOVA with Tukey’s multiple-comparison test [*, *P < *0.01]). (D) Representative photomicrographs of hematoxylin and eosin (H&E)-stained (upper panel) and immunohistochemically (IHC) stained (lower panel) lung sections from mice infected with the indicated viruses at 3 dpi. In IHC-stained tissues, the viral antigen is stained deep brown on a hematoxylin-stained background.

BALB/c mice were inoculated intranasally with serial dilutions of the four reassortants with H5N1-H9N2 hybrid phenotypes (#2, #7, #12, and #59 viruses) or with the H5N1-wt and #64 viruses, as controls, and monitored daily for weight loss and survival. With an inoculation dose of 10 FFU/mouse, the H5N1-wt virus caused no clinical effect during the 14-day observation period (Fig. 6A). In contrast, the #7 and #59 viruses caused drastic weight loss, and the #2, #12, and #64 viruses caused slight to moderate, but clearly detectable, weight loss. One of five mice infected by the #64 virus died at 11 days postinfection (dpi), two of five mice infected by the #2 and #12 viruses died by 12 dpi, and all five mice infected by the #7 and #59 viruses died by 12 dpi (Fig. 6B). The #59 virus had the highest pathogenicity in mice, with a 74.2-fold lower MLD_50_ than H5N1-wt.

We next investigated replication of the reassortants in the lungs of infected mice. Mice were inoculated intranasally with 10 FFU virus, and lungs were collected at 3 and 6 dpi for virus titer assays. Virus titers in the mouse lungs were in agreement with their replication in human cells *in vitro* (Fig. 3 and 5). In particular, the #7, #59, and #64 viruses produced virus titers that were >3 logs higher in mouse lungs at 3 and 6 dpi than did the H5N1-wt virus (Fig. 6C). Also, the #2 and #12 viruses replicated moderately in the lungs, with virus titers at 3 and 6 dpi that were up to 2 logs higher than those for than H5N1-wt.

The lungs of infected mice were collected at 3 dpi and examined by histopathology. H5N1-wt caused only a slight inflammatory response in the lungs of infected mice (Fig. 6D, upper panel). In contrast, the #7, #59, and #64 viruses induced severe bronchiolar necrosis and alveolitis, characterized by hemorrhage, dropout of epithelial cells, and infiltration of inflammatory cells. The #2 and #12 viruses caused light to moderate pneumonia in infected mice. The degree of inflammation generally corresponded to the amount of H5 antigen detected by immunohistochemistry in the alveolar areas of the lungs (Fig. 6D, lower panel).

**FIG 7:**
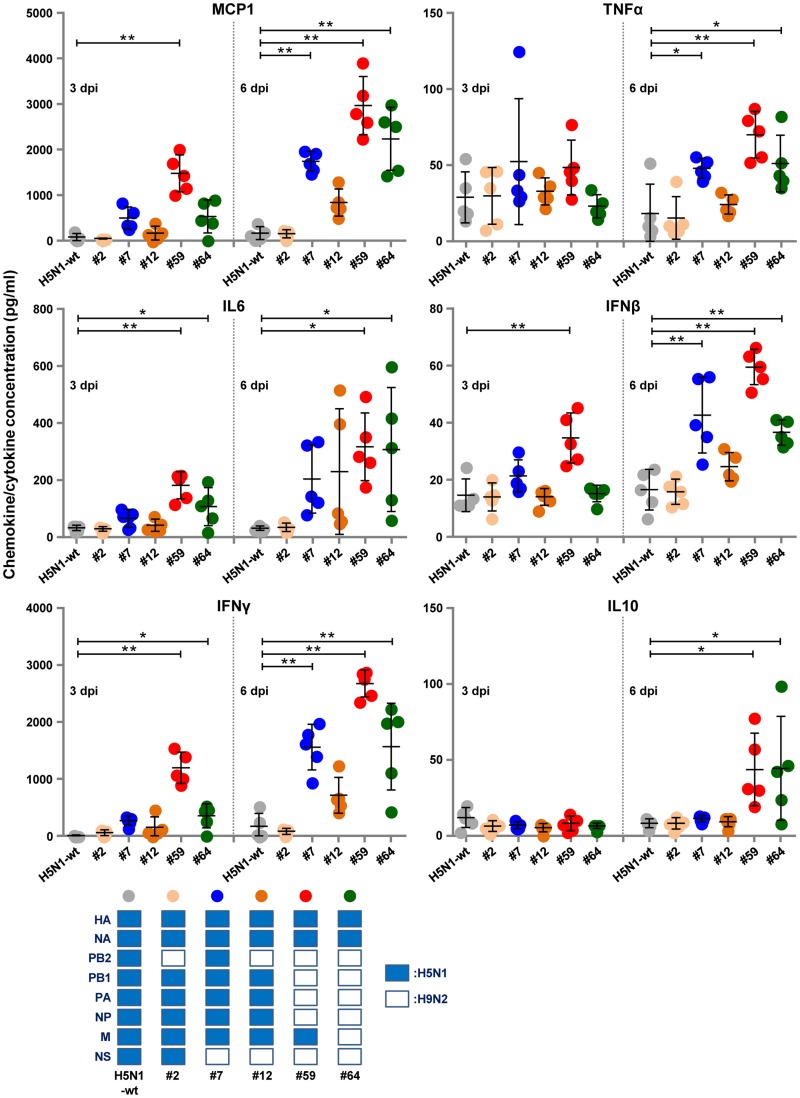
Chemokine/cytokine responses in the lungs of mice infected with 10 FFU of the indicated H5N1-H9N2 reassortants. The concentrations of the indicated chemokines and cytokines in the lungs of mice at 3 and 6 dpi were measured by ELISA. Each bar marks the concentration in an individual mouse. Asterisks indicate chemokine/cytokine levels significantly higher than those of H5N1-wt virus (ANOVA with Tukey’s multiple-comparison test [**, *P < *0.01; *, *P < *0.05]).

Chemokine/cytokine response analyses showed that the #7, #59, and #64 viruses induced appreciably strong immune responses in the lungs of infected mice. These three viruses induced significantly higher levels of monocyte chemoattractant protein 1 (MCP1), gamma interferon (IFN-γ), tumor necrosis factor alpha (TNF-α), and IFN-β at 6 dpi (Fig. 7). The #59 and #64 viruses also evoked significantly higher levels of interleukin-6 (IL-6) at both 3 and 6 dpi, IFN-γ at 3 dpi, and IL-10 at 6 dpi. This response was also characterized by high levels of MCP1 and IFN-β at 3 dpi by the #59 virus. These results suggested that efficient replication of the reassortants in human cells led to high progeny virus titers and to strong immune responses in infected mouse lungs, both of which generally produce high rates of lethality *in vivo*.

It was unexpected that the #7 virus had higher mouse lethality than the #64 virus, since the #7 virus titer in mouse lungs was lower than or similar to that of the #64 virus (Fig. 6B and C). To examine the factor(s) underlying this result, we investigated replication of the reassortants in the brains of infected mice. Of the six viruses in this study, a high progeny virus titer was only detected in the brains of mice infected by the #7 virus (Fig. 6E). In addition, mice inoculated with the #7 virus produced neurological symptoms beginning 6 dpi, which was the time postinfection of the onset of sudden weight loss and death in #7 virus-infected mice. No specific pattern of chemokine/cytokine response could be detected in mouse lungs infected with the #7 virus (Fig. 7). These results indicated that the #7 virus induced pneumonia and intercurrent neurological disorders due to its extrapulmonary extension to the brain, which eventually led to high lethality in mice.

Taken together, our data suggested that the H5N1-H9N2 reassortants in this study, which were constructed from H5N1 and H9N2 viruses isolated from birds, could acquire higher virulence in mice than the parental H5N1 and H9N2 viruses.

### Virion morphology of the reassortants.

**FIG 8:**
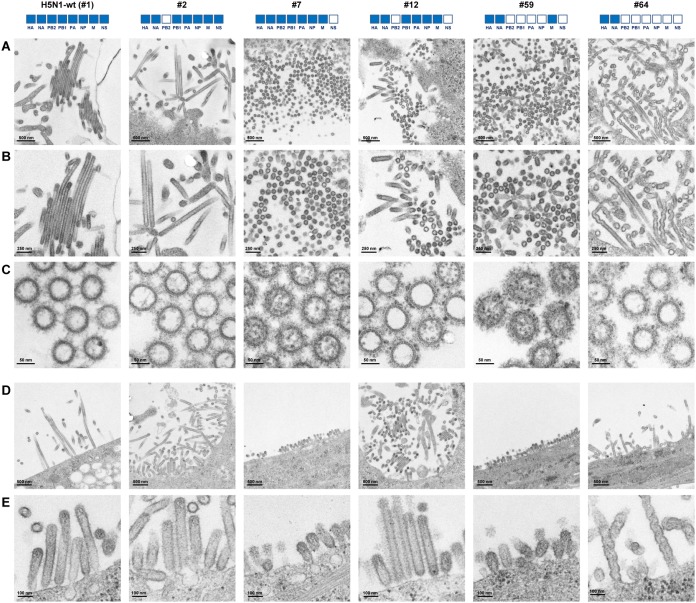
Virion morphology of H5N1-H9N2 reassortants. (A) Representative transmission electron micrographs of thin sections of the indicated virions released from infected MDCK cells at low magnification. (B) Magnified views of panel A. (C) Additional views of the indicated virions in transverse sections, in which viral ribonucleoproteins were observed. (D) Representative transmission electron micrographs of the indicated virions budding from the surface of infected MDCK cells at low magnification. (E) Additional views of virions budding from the cell surface at high magnification.

The influenza virus particle is pleomorphic, appearing as spherical and filamentous virions (20–22), but little is known about the functional significance of these morphologies. To investigate the virion morphology of the reassortants, MDCK cells were infected with reassortants with H5N1-H9N2 hybrid phenotypes (i.e., #2, #7, #12, and #59 viruses) and with the H5N1-wt and #64 viruses. At 18 hpi, the progeny virus particles released from host cell membranes were examined by transmission electron microscopy. H5N1-wt, #2, and #64 progeny virus particles were morphologically filamentous, with a helical filamentous shape for the #64 viruses, and the #12 virus produced a mixed population of shorter filamentous and spherical particles (Fig. 8A and B). In contrast, the #7 and #59 virus particles were predominantly spherical, with a few cup-shaped particles forming a rosette-like configuration in the #59 viruses. Ribonucleoproteins were sometimes observed within virus particles, most frequently within the smaller spherical virus particles (Fig. 8C). To further investigate the variation in virion morphology, virions budding from the infected cell surface were examined by electron microscopy at different magnifications. Most H5N1-wt, #2, and #64 virus particles budding from the cell surface were elongated or filamentous, with some filamentous bundles, and #12 virus particle were a mixture of short filamentous, ovoid and/or spherical particles (Fig. 8D and E). In contrast, #7 and #59 virus particles budding from the cell surface were primarily spherical. In total, these results indicated that most #7 and #59 virus particles were spherical, suggesting efficient release of these virus particles from infected cells.

## DISCUSSION

The cocirculation of H5N1 and H9N2 viruses in Egypt provides opportunities for coinfections to produce reassortant viruses. Using reverse genetics, we found high genetic compatibility of H5N1-H9N2 reassortants, resulting in higher virulence of reassortants in human cells and mice than the parental H5N1 and H9N2 viruses. To our knowledge, this study presents the first broad-spectrum data on the emergence potential of H5N1-H9N2 reassortants with distinct phenotypes. Previous studies using reverse genetics in the genetic background of an AI virus and of a seasonal influenza virus showed various degrees of genetic compatibility between the two viruses, with occasional high pathogenicity in humans (23–26). In contrast, our study showed that reassortment between the AI viruses, even two subtypes of a bird species, could generate hybrid viruses with significantly high public health risk for humans.

A coinfection study of A/chicken/Egypt/AR236/2015 (H5N1) and A/chicken/Egypt/AR755/2013 (H9N2) in embryonated chicken eggs reported the recovery of five genotypes of reassortants (16). Our results are in agreement with that study and showed the recovery of viable reassortants of all 63 genotypes (Fig. 1), highlighting the applicability of our reverse-genetics approach for studies of influenza virus reassortants. Collectively, the recovery of five genotypes of reassortants from coinfections (16) and the data presented here of the 63 genotypes in our reverse genetics study indicated the emergence potential of H5N1-H9N2 reassortants in Egypt.

Nonetheless, no natural H5N1-H9N2 reassortant has been detected in Egypt thus far. It has been suggested that this has been mainly due to insufficient surveillance, with only a small number cases being analyzed by full genome sequencing, and not due to genetic incompatibility between the subtypes (16). Our results, indicating high genetic compatibility between the two virus subtypes, supported that suggestion. However, our results also showed that replication of most genotypes (55 of 63) was lower than that of the parental H5N1 virus in avian cells (Fig. 3A). This indicated negative selective pressure in birds to generate H5N1-H9N2 reassortants, which may be one of the reasons for the failure to detect such reassortants in the field. However, seven of the genotypes in this study may emerge in birds under neutral selective pressure (i.e., the #2, #3, #5, #7, #12, #21, and #43 viruses) and one may emerge under positive selective pressure (i.e., the #59 virus) in nature (Fig. 3A and 5A). Conversely, replication of the majority of the reassortants in human cells was higher than that of the parental H5N1 virus at both 37°C (42 of 63 reassortants) and 33°C (46 of 63 reassortants) (Fig. 3B, 3C, 5B, and 5C), suggesting that coinfection by H5N1 and H9N2 viruses in humans or other mammalian species may generate hybrid viruses with high infectivity and significant public health risk. Taken together, our comprehensive data indicated that reassortants between contemporary H5N1 and H9N2 viruses in Egypt could be transmitted efficiently to humans once they emerge in the field, although they are not likely to emerge frequently in birds.

Our data showed that the H9N2 NS gene generally produced increased replication of the reassortants to levels comparable to the H9N2-wt in human cells but not in avian cells, whereas the H9N2 PB2 gene produced a smaller increase in replication (Fig. 3). On the other hand, the H5N1 M gene contributed to the maintenance of reassortant replication in avian cells at levels comparable to H5N1-wt. Reassortants that replicated well in both avian and human cells in this study (i.e., #2, #7, #12, and #59 viruses) contained the H5N1 M gene and one or both of the H9N2 NS and PB2 genes (Fig. 5). These results indicated that the H5N1 M gene, together with the H9N2 NS and/or PB2 gene, may enable the emergence of reassortants with an H5N1-H9N2 hybrid phenotype.

The NS gene encodes NS1 and NEP, which are produced by pre-mRNA splicing (27). NS1 is a multifunctional protein that mainly plays key roles as an IFN antagonist in inhibition of the host immune response (28–30). The #7, #12, #59, and #64 viruses carrying the H9N2 NS gene had an increase in both progeny virus yield and virulence (Fig. 6) and also in the chemokine/cytokine response in infected mice (Fig. 7) compared to the parental H5N1 virus. This implied that H9N2 NS increased viral replication in human cells by a mechanism(s) other than by an IFN antagonist effect. There were no amino acid differences in the known virulence markers in both NS1 and NS2 of the H5N1 and H9N2 viruses that might account for the higher virulence in mice by reassortants containing the H9N2 NS gene (i.e., NS1 [residues 42, 80 to 84, 92, 103, 106, and 189 and the PDZ domain ligand motif in the C terminus] and NS2 [residues 31 and 56]), as has also been reported (7). Several studies reported additional NS gene roles in supporting viral growth in multiple steps in the virus life cycle, including viral replication, mRNA stabilization, and translation (31–33). Thus, we assumed that H9N2 NS acted on one of these steps in human cells. Our results in this study indicated a possible contribution by H9N2 NS in human adaptation of AI viruses due to reassortment events, although the underlying mechanism(s) remains unknown.

Spherical morphology, rather than filamentous morphology, of the influenza viruses in this study seemed to be at least partially correlated with more efficient viral replication in human cells and mice (Fig. 5, 6, and 8), implying that spherical morphology may have facilitated virus shedding and/or infection of the H5N1-H9N2 reassortants. Spherical virus particles have been reported to be associated with a higher output of influenza viruses (34–36). These results are in agreement with a recent study showing that reassortment was associated with a change from filamentous to spherical morphology, which led to extrapulmonary viral spread and more severe pathogenicity in animals (34). This suggested that the spherical phenotype is better adapted to replication of H5N1-H9N2 reassortants in human cells *in vitro* and in mice *in vivo*. Virion formation is a multifaceted process (20–22). There are a number of viral factors that may have affected the morphology of the reassortant viruses in this study (i.e., M, NS, and NP genes) (20–22, 37, 38). Spherical virion morphology was seen in reassortants with the H5N1 M and H9N2 NS genes (i.e., #7, #12, and #59) in this study, whereas filamentous morphology was seen in reassortants with homologous combinations of the M and NS genes from the H5N1 or H9N2 viruses (i.e., H5N1-wt, #2, and #64) (Fig. 8). This implied a possible association of the heterologous H5N1 M and H9N2 NS genes in the formation of spherical virions by reassortants. However, the determinant(s) of morphological changes and the significance of morphology in viral fitness in this study remain largely unclear, and further data are needed.

In this study, we characterized all 63 possible reassortant genotypes carrying the H5N1 surface gene segments and combinations of the H5N1 and H9N2 internal gene segments. H5N1 clade 2.2.1.2, including CL69, generally has an HA multibasic cleavage site (1); an HA-133Δ/I155T mutation (H3 numbering) that provides dual tropism for both α2,3 Sia, and α2,6 Sia (17); and a representative human-adaptation PB2-E627K mutation (1, 2). These genetic traits define, at least partially, the high infectivity and pathogenicity of viruses in this clade for humans. However, the contemporary H9N2 G1-B lineage, including CL42, has an HA-Q226L mutation that produces increased binding affinity for α2,6 Sia (7, 18) and a PB2-E627V mutation that recently has been reported to increase AI virus replication in human cells (39). Indeed, our analysis showed that the two subtype strains (i.e., CL69 and CL42) had similar levels of α2,6 Sia binding affinity (Fig. 1A and B) and substantial replication ability in human cells *in vitro*, suggesting that these strains may have a similar high potential for transmission from birds to infect humans. However, the presence of a monobasic cleavage site in the HA in Egyptian H9N2 viruses can produce relatively limited viral replication in mammals *in vivo* (8, 40). In fact, H9N2-wt had considerably lower virulence in mice than H5N1-wt: the MLD_50_ values for H9N2-wt and H5N1-wt were 3.2 × 10^4^ and 5.1 × 10^1^ FFU, respectively (Fig. 1C and D). In contrast, the #64 virus, which carried HA and NA genes from H5N1-wt and all six internal genes from H9N2-wt, was virulent in mice with a lower MLD_50_ (3.2 × 10^1^ FFU) than H9N2-wt (Fig. 6), indicating that the H9N2 HA and NA genes produced limited replication and virulence in mammals. Thus, we anticipate that reassortants carrying H9N2 surface genes may have lower or possibly similar pathogenicity for humans compared to reassortants with H5N1 surface genes. Nevertheless, reassortants carrying H9N2 surface genes may cause asymptomatic or less symptomatic infections in poultry and humans. This would allow human cases to go unreported and increase the opportunity for virus transmission to humans, with subsequent reassortment or mutation, to emerge as a novel virus with high public health risk. Therefore, the possible emergence of influenza virus reassortants carrying both H5N1 and H9N2 surface genes needs to be carefully monitored.

In conclusion, our analyses indicated a substantial emergence potential of influenza virus reassortants derived from the H5N1 and H9N2 viruses currently cocirculating in Egypt, as well as the possibility of their high public health risk for humans relative to the parental H5N1 and H9N2 viruses. Cocirculation of the two influenza virus subtypes in birds may accelerate the emergence of novel viruses that may be a public health risk. Our results underscore the necessity for enhanced influenza virus surveillance strategies to monitor reassortment events in nature and reduce the public health threat posed by the H5N1 and H9N2 viruses cocirculating in Egypt.

## MATERIALS AND METHODS

### Ethics statement.

All animal experiments were conducted in compliance with Japanese legislation (Act on Welfare and Management of Animals, 1973, revised in 2012) and guidelines under the jurisdiction of the Ministry of Education, Culture, Sports, Science, and Technology of Japan (Fundamental Guidelines for Proper Conduct of Animal Experiment and Related Activities in Academic Research Institutions, 2006). Animal care, housing, feeding, sampling, observation, and environmental enrichment were approved by the Animal Experiment Committee of the Kyoto Prefectural University of Medicine (approvals M29-554 and M30-60).

### Biosecurity and biosafety.

All experiments with live H5N1 and H9N2 viruses were performed at enhanced biosafety level 3+ (BSL3+) at the Kyoto Prefectural University of Medicine. All studies with recombinant DNAs were conducted under the applicable laws in Japan and approved by the Biological Safety Committee of Kyoto Prefectural University of Medicine (approvals 25-2 and 30-104) after risk assessments were conducted by the Living Modified Organisms Committee of Kyoto Prefectural University of Medicine and, when required, by the Ministry of Education, Culture, Sports, Science, and Technology of Japan.

Only authorized personal that have received appropriate training can access the BSL3+ facility. All personnel working at the BSL3+ facility wear a disposable, protective, FFP3 facemask and multiple pairs of gloves. Furthermore, all personnel conducting this study were vaccinated against seasonal and H5N1 influenza viruses. The facility is secured by procedures recognized as appropriate by the institutional biosafety officers at Kyoto Prefectural University of Medicine and by Japan government inspectors. Antiviral drugs are directly available to further mitigate risks upon incidents.

Furthermore, in this study, a mouse infection study was performed after observing no increase in viral growth by the reassortants used in cell cultures compared to previously published studies (2, 16, 41–45).

### Cells and viruses.

Calu-3 and DF-1 cells were obtained from the ATCC (https://www.atcc.org/), and MDCK and 293T cells were obtained from the RIKEN BioResource Center Cell Bank (http://www.brc.riken.jp/lab/cell/english/). The cells were maintained in Dulbecco modified Eagle medium (DMEM) with 10% fetal calf serum at 37°C in a humidified atmosphere of 95% air and 5% CO_2_. Influenza viruses A/chicken/Egypt/CL69/2013 (CL69) (H5N1) and A/chicken/Egypt/CL42/2013 (CL42) (H9N2) were isolated from diseased chickens in the delta area of Egypt in November 2013.

### Virus preparation.

Isolated and recombinant avian influenza viruses were grown in 10-day-old embryonated eggs with one or two passages. The allantoic fluids were then harvested and stored at –80°C. For subsequent studies, allantoic fluids were precleared by centrifugation at 3,000 rpm for 20 min and filtration through 0.45-μm-pore-size filters, and viruses were then purified by centrifugation at 25,000 rpm for 2 h through a 20 to 60% sucrose gradient. After collecting the virus-containing fractions, the viruses were suspended in phosphate-buffered saline (PBS), layered on a 20% sucrose cushion and pelleted by centrifugation at 25,000 rpm for 2 h. Virus pellets were resuspended in PBS and aliquots were stored at −80°C as working stocks. Virus titers were assayed by focus-forming assays (41).

### Phylogenetic analysis.

The sequences of viral gene segments from reference H5N1 and H9N2 strains isolated in 1997 to 2015 were obtained from the GISAID database (https://www.gisaid.org). Phylogenetic analysis was performed using MEGA4 software for the neighbor-joining method, with the nucleotide sequences of representative H5N1 and H9N2 strains, including the CL69 and CL42 strains isolated in this study. The confidence levels for the phylogenetic trees were calculated by performing 1,000 bootstrap replicates.

### Generation of viruses by reverse genetics.

Recombinant viruses were generated with a plasmid-based reverse genetics system in A/chicken/Egypt/CL69/2013 (CL 69) (H5N1) and A/chicken/Egypt/CL42/2013 (CL42) (H9N2) genetic backgrounds as previously described (17, 41, 44). All constructs were completely sequenced to ensure the absence of unwanted mutations.

### Receptor binding assays.

Receptor binding specificity was analyzed by a solid-phase direct binding assay (44, 45) with α2,3 and α2,6 sialylglycopolymers. Serial dilutions of each sialylglycopolymer were prepared in PBS, and a 100-μl sample was added to each well of 96-well microtiter plates (Polystyrene Universal-Bind Microplates; Corning). The plates were then irradiated with 254-nm UV light for 10 min, and each well was washed three times with 250 μl of PBS. Each well was blocked with 200 μl of SuperBlock blocking buffer (Thermo Scientific) at room temperature for 1 h. After a wash with ice-cold PBS containing 0.1% Tween 20 (PBST), a solution containing influenza viruses (16 HAU in PBST) was added to each well, and the plates were incubated at 4°C for 12 h. After five washes with ice-cold PBST, mouse anti-NP antibody (against the influenza virus NP protein) was added to each well, and the plates were incubated at 4°C for 2 h. The wells were then washed five times with ice-cold PBST and incubated with peroxidase-conjugated goat anti-immunoglobulin (Histofine Simple Stain MAX-PO; Nichirei) at 4°C for 2 h. After five washes with ice-cold PBST, 100 μl of SureBlue reserve peroxidase substrate was added to each well and, after incubation at room temperature for 5 min, the absorbance at 630 nm was measured. Each data point represents the means ± the standard deviations (SD) of three experiments, each performed in duplicate.

### Infection of cultured cells.

DF-1 and Calu-3 cells (90% confluent in collagen-coated 24-well plates) were infected in triplicate with the indicated viruses at MOIs of 0.003 and 0.03, respectively. After 1 h at 37°C, the cells were washed with PBS and maintained in DMEM/F-12 containing 0.2% bovine serum albumin at 33 or 37°C. Acetylated trypsin (2 μg/ml; Sigma-Aldrich) was also added to the cell cultures for the propagation of H9N2-wt. At the indicated times postinfection, the virus titers in the cell culture supernatants were assayed by focus-forming assays.

### Minigenome assays.

293T cells were transiently transfected as described previously (41, 46), with the following plasmid mixture: polymerase II-driven pcXN2 plasmids each expressing a PB2, PB1, PA, or NP gene from either CL69 or CL42, as well as a human polymerase I-driven pPol luciferase plasmid. Cells were transfected with the plasmid mixture using TransIT-LT1 (Mirus) and incubated at 33 or 37°C. At 24 h posttransfection, the cells were lysed, and the luciferase activity was determined by a dual-luciferase assay system (Promega) according to the manufacturer’s instructions.

### Experimental infections in mice.

Five-week-old female BALB/c mice (Japan SLC), under mixed anesthesia (medetomidine-butorphanol-midazolam), were intranasally inoculated with 50-μl samples of 1 to 10^5^ FFU virus in PBS. The body weights and survival of the mice were monitored daily for 14 days. Mice that lost more than 30% of their original weight were humanely euthanized. Lungs and brains of mice infected with 10 FFU virus were collected 3 and 6 dpi, and virus titers were assayed by focus-forming assays. Enzyme-linked immunosorbent assays (ELISAs) were performed on lung homogenates using CCL2 (MCP1), IL-6, IL-10, TNF-α, IFN-γ, and IFN-β Quantikine kits (R&D Systems). For histopathology analysis, the lungs of mice infected with 10 FFU of virus were collected at 3 dpi, fixed in 4% buffered paraformaldehyde, embedded in paraffin, cut into 5-μm sections, stained with hematoxylin and eosin, and examined by light microscopy. Immunohistochemical staining of the viral antigen was performed, as described previously (17, 41), using a monoclonal antibody (C43) specific for influenza A virus nucleoprotein and a Mouse-on-Mouse (MOM) peroxidase kit (Vector Laboratories) with diaminobenzidine as the chromogen and hematoxylin as the counterstain. Unrelated antibodies were used in place of the primary antibody as controls.

### Electron microscopy.

MDCK cells were infected with the indicated viruses at an MOI of 3. After 18 h of incubation, the cells were fixed with 2% paraformaldehyde and 2% glutaraldehyde in 0.1 M phosphate buffer (PB; pH 7.4) for 30 min at 4°C, and with 2% glutaraldehyde in 0.1 M PB overnight at 4°C. The samples were then washed three times with 0.1 M PB for 30 min each, and postfixed with 2% osmium tetroxide (OsO_4_) in 0.1 M PB at 4°C for 1 h. After fixation, the samples were dehydrated and embedded in resin (Quetol-812; Nisshin EM Co.), and ultrathin 70-nm sections were cut with a diamond knife using an ultramicrotome (Ultracut UCT; Leica). The samples were mounted on copper grids and stained with 2% uranyl acetate, followed by secondary staining with a lead stain solution (Sigma-Aldrich Co.). After sample preparation, the grids were observed with a transmission electron microscope at 100 kV (JEM-1400Plus; JEOL, Ltd.). Digital images were captured by a charge-coupled device camera (EM-14830RUBY2; JEOL, Ltd.) and visualized using Adobe Photoshop software (Adobe Systems, Inc.).

### Statistical analysis.

Statistical analysis was carried out using Prism v5 software (GraphPad Software, Inc.). Statistically significant differences of a group of 64 virus pairs were determined by analysis of variance (ANOVA) using a Tukey’s multiple-comparison test.

### Accession number(s).

Recombinant viruses were generated with a plasmid-based reverse genetics system as described above. The sequences have been deposited in the GenBank database under accession numbers LC379955 to LC379962 for the CL69 strains and LC379963 to LC379970 for the CL42 strains.

## Supplementary Material

Supplemental file 1
