# Supplementary material for: Genetic Compatibility of Reassortants between Avian H5N1 and H9N2 Influenza Viruses with Higher Pathogenicity in Mammals
Source: J Virol. 2019 Feb 5;93(4):e01969-18. doi: 10.1128/JVI.01969-18 (PMC6363993; doi:10.1128/JVI.01969-18)
Supplement: Supplemental file 1 [file 87044705d0e58a8fcfd75f4326ee934a_JVI.01969-18-s0001.pdf]

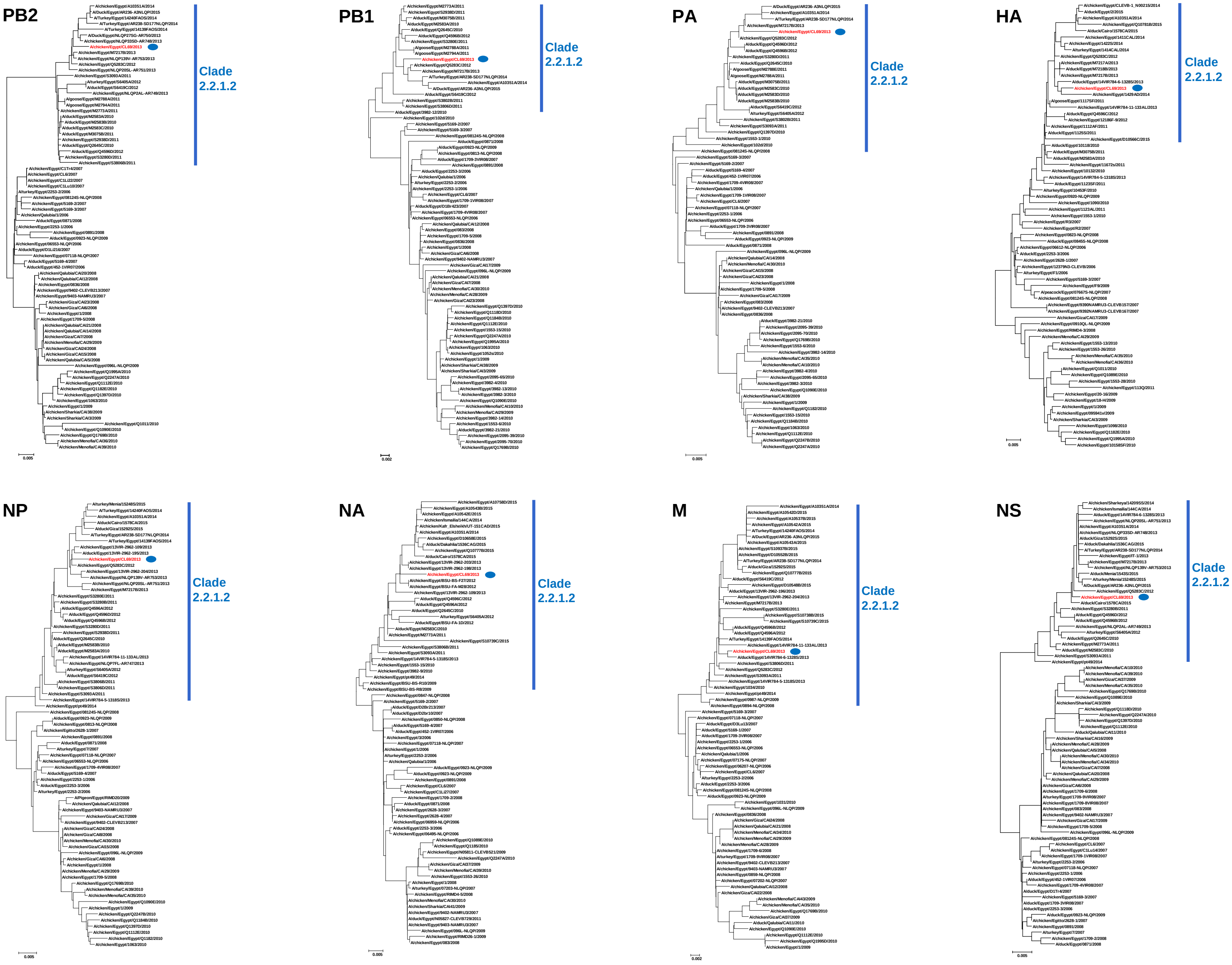


**Fig. S1. Phylogenetic trees of the genes of H5N1 viruses isolated in Egypt.** The phylogenetic trees were reconstructed from nucleotide sequences of the Egyptian reference strains deposited in the GISAID database and of the CL69 virus isolated in this study (in red font and marked with a circle in each tree).

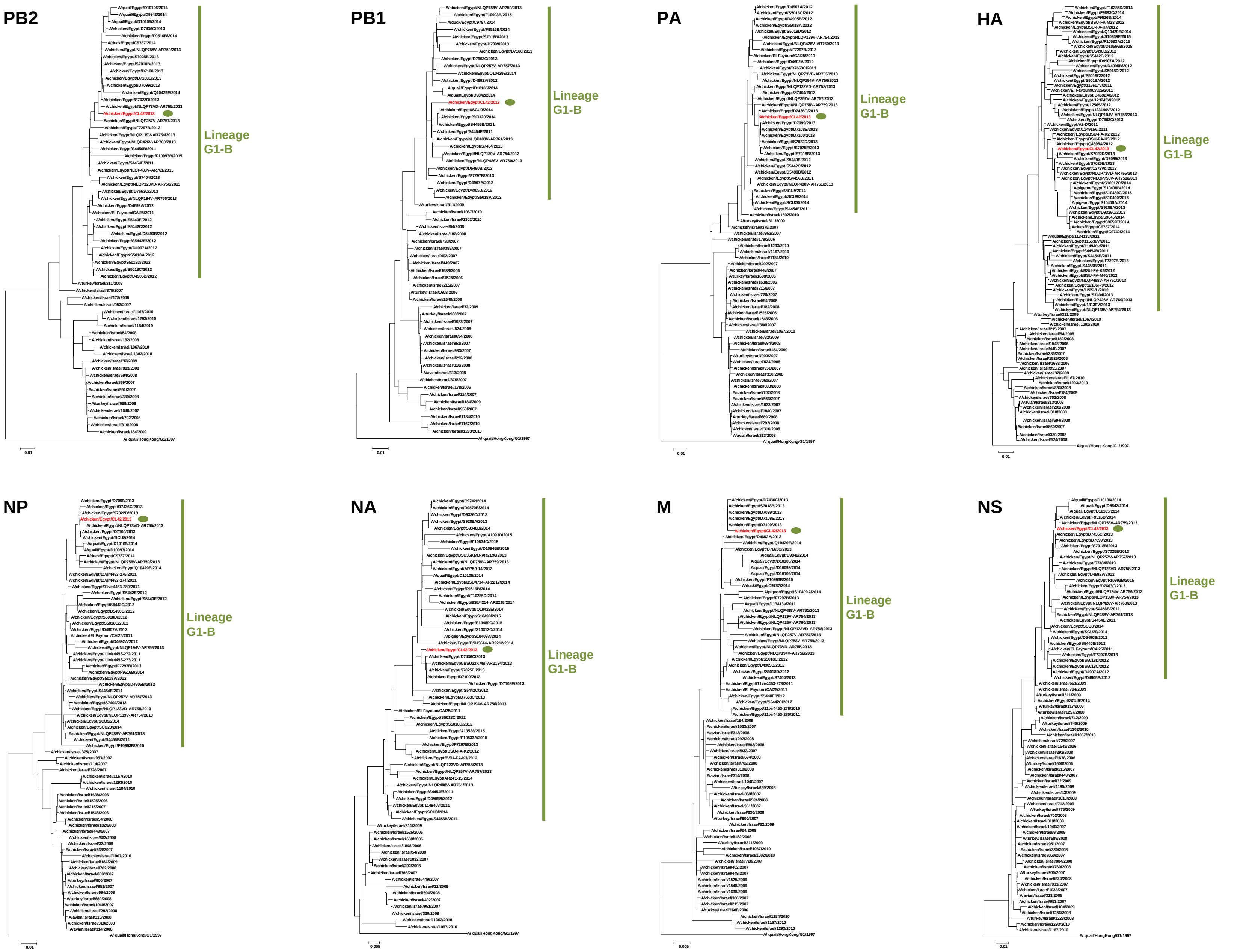


**Fig. S2. Phylogenetic trees of the genes of H9N2 viruses isolated in Egypt.** The phylogenetic trees were reconstructed from nucleotide sequences of the Egyptian reference strains deposited in the GISAID database and of the CL42 virus isolated in this study (in red font and marked with a circle in each tree).
